# The Relationship Between Individual Characteristics and Interest in Using a Mobile Phone App for HIV Self-Management: Observational Cohort Study of People Living With HIV

**DOI:** 10.2196/mhealth.7853

**Published:** 2017-07-27

**Authors:** Robert James Lucero, Jemima A Frimpong, Elizabeth A Fehlberg, Ragnhildur I Bjarnadottir, Michael T Weaver, Christa Cook, Francois Modave, Mobeen H Rathore, Jamie P Morano, Gladys Ibanez, Robert L Cook

**Affiliations:** ^1^ Department of Family, Community, and Health Systems Science College of Nursing University of Florida Gainesville, FL United States; ^2^ Carey Business School John Hopkins University Baltimore, MD United States; ^3^ Division of Research on Healthcare Value, Equity, and the Lifespan RTI International Research Triangle Park, NC United States; ^4^ Department of Health Outcomes and Policy College of Medicine University of Florida Gainesville, FL United States; ^5^ College of Medicine University of Florida Jacksonville, FL United States; ^6^ Morsani College of Medicine University of South Florida Tampa, FL United States; ^7^ Department of Epidemiology Robert Stempel College of Public Health and Social Work Florida International University Miami, FL United States; ^8^ Department of Epidemiology Colleges of Medicine and Public Health University of Florida Gainesville, FL United States

**Keywords:** telemedicine, self-care, HIV

## Abstract

**Background:**

The human immunodeficiency virus (HIV) continues to be a major health issue in the United States, and an estimated 1.2 million people in the United States are living with HIV. As part of Healthy People 2020, the Office of Disease Prevention and Health Promotion has targeted the persistent demographic and geographic disparities in HIV prevalence and management. Preliminary evidence suggests that mobile health technology (smartphone apps) may be a promising way to support HIV self-management among vulnerable populations of people living with HIV (PLWH) who lack access to appropriate health care services.

**Objective:**

This study examines the association between individual characteristics of PLWH and level of interest in using a free mobile phone app for HIV self-management.

**Methods:**

This study was conducted using cross-sectional survey data collected in the Florida Cohort Study between 2014 and 2016 (N=766). Associations between individual characteristics of PLWH and level of interest in using a free mobile phone app for HIV self-management were examined using bivariate analysis and logistic regression.

**Results:**

Overall, 85.5% (655/766) of respondents were interested in using a free mobile phone app that supports HIV self-management. Participants expressed the highest interest in app functions that facilitate communication with health care providers (568/740, 76.8%) or help to identify relevant health care services (556/745 74.6%). Age (OR 0.959, 95% CI 0.936-0.982), education (OR 1.281, 95% CI 1.027-1.598) and disability or inability to work (OR 0.296, 95% CI 0.145-0.606) were all significantly associated with being interested in using a free mobile phone app for HIV self-management.

**Conclusions:**

This study indicates that a majority of PLWH are interested in using a free mobile phone app to self-manage their condition. The findings can inform the development of mobile phone apps that support effective HIV self-management.

## Introduction

Human immunodeficiency virus (HIV) continues to be a major health issue in the United States, and an estimated 1.2 million people are living with the disease [1]. Demographic and geographic disparities exist in the incidence and prevalence of HIV. HIV disproportionately affects African Americans and Hispanics/Latinos. African Americans represent only 12% of the US population but account for 44% of nationwide HIV diagnoses [1]. Similarly, Hispanics/Latinos represent 17% of the US population but account for an estimated 23% of new HIV diagnoses [1]. Moreover, Latinos are the fastest growing segment of the population in the United States. These disparities are even more pronounced in rural areas [2,3], particularly in the rural South [4]. The southern states in general account for 44% of all people living with HIV (PLWH) in the United States even though they represent only a third of the nation’s overall population. In the southern states, African Americans account for 54% of new HIV diagnoses [4]. The state of Florida ranks second in the country for highest prevalence of HIV infection, with a rate of 594.8 cases per 100,000 people [5]. In Florida, those living outside of metropolitan areas account for 18.6% of all Florida residents living with HIV/AIDS, and African Americans represent 48% of PLWH in the state despite only representing 15% of the Florida population [5]. Therefore, there is a great need for better understanding demographic and geographic disparities in this area and how best to address them.

While HIV was previously a common fatal disease, the advent of antiretroviral therapy (ART) has transformed it to a chronic condition, allowing people to live long lives with HIV infection [6]. Successful HIV treatment depends on attending regular HIV care appointments and adherence to medications. In 2011, only 40% of persons living with HIV were engaged in medical care for their condition, 37% were prescribed ART, and only 30% were ART adherent to the point of achieving viral suppression [7]. For most patients, near perfect adherence is necessary to achieve individual and population health benefits of ART [8,9]. While strides have been made in the development of self-management programs and resources, the effectiveness and feasibility of such programs to improve outcomes and promote health has not been well established for PLWH [6,10].

Mobile health (mHealth) interventions have emerged as a promising tool to support disease self-management among PLWH from all demographic groups and geographic areas [11-16]. Ownership of smartphones and other mobile devices has grown rapidly with an estimated 68% of Americans and 62% of smartphone owners reporting using their devices to seek health information [17]. Mobile health technologies have shown promise in improving patient communication with their provider, providing education, and supporting management of various chronic conditions including diabetes, cardiovascular disease, and HIV [18-21]. However, for mHealth interventions to be effective, they need to be developed and optimized with the needs of PLWH in mind [22,23]. Because little is known about the level of interest in using phone apps for disease self-management among PLWH, this study aimed to examine (1) PLWH’s preferences for functions in a free mobile phone app for HIV self-management and (2) associations between individual characteristics of PLWH and level of interest in using a free mobile phone app for HIV self-management.

## Methods

### Recruitment

This study was conducted using cross-sectional survey data collected in the Florida Cohort Study between 2014 and 2016. The Florida Cohort Study uses a convenience-sampling frame across several public health settings in Florida to recruit PLWH and collect information about demographic, behavioral, and social factors affecting health outcomes. Any person with HIV older than 18 years of age was eligible to participate in the study. Participants were recruited from a collaborative network of county health department and community clinics throughout Florida, including sites at Lake City, Gainesville, Tampa, Orlando, Sanford, Ft. Lauderdale, and Miami. After written informed consent was obtained, anonymous surveys were self-administered by cohort participants using Research Electronic Data Capture, a secure, Web-based app. Surveys were completed in English or Spanish depending on the preference of the participant. These surveys took approximately 30 to 45 minutes to complete and respondents were provided $25 in compensation in the form of a gift card. The University of Florida, Florida International University, and the Florida Department of Health institutional review boards approved the Florida Cohort Study.

### Data Collection

The dependent variable in this study was a binary measure we created that indicates interest in a free mobile phone app for HIV self-management. Level of interest in functions that support self-management using mobile technology was determined using the following set of survey questions: “If available and free, how often would you use a phone app to help you: (1) identify health services relevant to you, (2) track changes in your mood and emotions, (3) provide tips to improve your health, based on information about you, (4) manage alcohol and drug use behavior, (5) communicate with your doctor or clinic, (6) remember to take your medication, or (7) engage in social networking with other people with similar health conditions as you?” Possible answers for these 7 questions were never, rarely, about once a week, a few times a week, and daily. Interest was defined as any response choice other than never.

Individual characteristics that were analyzed included age and amount of schooling completed, which ranged from: (1) elementary school or below, (2) some high school, (3) high school graduate or general education diploma (GED), (4) some college or technical/trade school, (5) college or trade school graduate, or (6) graduate degree or professional degree after graduating college. Additional individual characteristics that were analyzed included sex at birth, ethnicity, race, being in a long-term partnership, sexual orientation, and employment status. Ethnicity was categorized as being Hispanic versus not Hispanic based on whether the respondent self-reported being of Hispanic/Latino origin or descent. Race was categorized as white, Black/African American, or other race. The category of other race included Native American, Asian, multiracial, and other responses by participants. Being in a long-term partnership was a binary variable that indicated marriage or living with a long-term partner versus the state of being divorced, widowed, separated, or never married/single. Sexual orientation was categorized as heterosexual, gay or lesbian, and other sexual orientation. The other sexual orientation category included options of bisexual, asexual, and other. Employment status was determined by asking respondents to select from types of current employment: employed for wages, self-employed, out of work for more than 1 year, out of work for less than 1 year, homemaker, student, retired, or unable to work/disabled. These employment statuses were collapsed into 3 categories: employed, unemployed, and unable to work/disabled. The employed category included all respondents that selected employed for wages or self-employed. The unemployed category included all of the remaining respondents except for those who were unable to work/disabled.

### Statistical Analysis

At the time of the analysis, we included all 766 participants in the Florida Cohort Study. Statistical analysis was performed using SAS software, version 9.4 (SAS Institute Inc). Univariate descriptive statistics were calculated including the mean, median, and range for continuous variables and counts and percentages for categorical variables. All variables were examined as categorical variables to determine bivariate relationships using chi-square analysis. Individual characteristics with *P<*.25 were included in the multivariable model [24]. Sex at birth was not included in the model (*P*=.49). After the bivariate relationships were determined, we evaluated whether age and amount of schooling completed could be included in the multivariable analysis as continuous variables. Specifically, to evaluate the linearity of age and amount of schooling completed, a Box-Tidwell approach was used by employing the natural log of the variables [25]. Based on the results of the Box-Tidwell approach, both age and amount of schooling completed were treated as linear continuous variables. For the multivariable analysis, logistic regression was used to calculate adjusted odds ratios (ORs) and the corresponding 95% confidence intervals.

## Results

The individual characteristics of respondents in the sample are presented in Table 1. A majority of the respondents were male (65.8%), with an average age of 46 years. Only 15.2% of the sample was of Hispanic ethnicity. African-Americans accounted for 59.1% of the sample, followed by whites, who accounted for 31.8% of the sample. On average, participants reported completing high school or obtaining a general education diploma. Nearly 8 out of every 10 participants indicated that they were not in a relationship. A majority (52.7%) of the respondents were heterosexual while slightly more than one-third of the sample (35.2%) were gay or lesbian. Almost half (48.7%) of the respondents were unable to work/disabled, 26.4% were unemployed, and 24.8% were employed at the time they completed the survey. Overall, 85.5% of respondents were interested in using a free mobile phone app that supports HIV self-management.

**Table 1:** Demographic characteristics (N=766).

Demographic variable		N	Frequency, n (%)	Mean (SD)	Median	Range
**Sex at birth**		766				
	Male		504 (65.8)			
	Female		262 (34.2)			
Age		760		45.99 (11.27)	48.00	19.00-77.00
**Ethnicity**		765				
	Hispanic		116 (15.2)			
	Not Hispanic		649 (84.8)			
**Race**		765				
	Other race^a^		70 (9.2)			
	Black/African American		452 (59.1)			
	White		243 (31.8)			
Schooling completed^b^		764		3.14 (1.15)	3.00	1.00-6.00
**In a long-term partnership^c^**		764				
	Yes		155 (20.3)			
	No		609 (79.7)			
**Sexual orientation**		744				
	Heterosexual		392 (52.7)			
	Gay or lesbian		262 (35.2)			
	Other sexual preference^d^		90 (12.1)			
**Employment status**		749				
	Employed		186 (24.8)			
	Unable to work/disabled		365 (48.7)			
	Unemployed		198 (26.4)			

^a^Other race includes Native American, Asian, multiracial, and other.

^b^School completed ranges from 1-6: 1=elementary school or below, 2=some high school, 3=high school graduate or general education diploma, 4=some college or technical/trade school, 5=college or trade school graduate, 6=graduate degree or professional degree after graduating college.

^c^Long-term partnership includes married or living with long-term partner.

^d^Other sexual preference includes bisexual, asexual, and other.

Respondents’ level of interest in using a free mobile phone app to support HIV self-management is shown in Table 2. Respondents had the highest interest in app functions that facilitate communication with their doctor or clinic (76.8%) and help to identify relevant health services (74.7%). Respondents were also interested in app functions that provide tips to improve health based on personalized information (67.7%) and supply reminders to take medication (60.7%). Nearly 3 out of 5 participants were interested in an app function that enabled social networking with individuals with similar health conditions. Participants had the least interest in app functions for tracking changes in mood or emotions (53.8%) and managing alcohol and drug use behavior (31.6%).

Table 3 shows the multivariate regression results. An increase in age (OR 0.959, 95% CI 0.936-0.982) and unable to work/disabled (OR 0.296, 95% CI 0.145-0.606) were significantly associated with lack of interest in using a free mobile phone app for HIV self-management. Conversely, greater educational attainment was positively associated (OR 1.281, 95% CI 1.027-1.598) with a high level of interest in using a free phone app to support HIV self-management.

**Table 2:** Interest in mobile phone app functions to support HIV self-management.

Mobile app function	N	Never (%)	Rarely (%)	About once a week (%)	Few times a week (%)	Daily (%)	Interested^a^ (%)	Not Interested^b^ (%)
Identify health services^a^	745	25.4	20.4	16.0	14.4	23.9	74.6	25.4
Track mood or emotions^b^	744	46.2	17.3	7.8	9.0	19.6	53.8	46.2
Provide health tips^c^	745	32.3	18.4	11.4	13.2	24.7	67.7	32.3
Manage alcohol and drug use^d^	741	68.4	11.2	4.7	4.3	11.3	31.6	68.4
Communicate with your doctor^e^	740	23.2	18.0	19.5	16.1	23.2	76.8	23.2
Remember to take your medication^f^	743	39.3	11.6	4.7	5.1	39.3	60.7	39.3
Engage in social networking^g^	741	40.8	15.1	9.6	11.5	23.1	59.2	40.8

^a^Considered interested if answered rarely, about once a week, a few times a week, or daily.

^b^Considered not interested if answered never.

^c^Identify health services relevant to you.

^d^Track changes in your mood or emotions.

^e^Provide tips to improve your health, based on information about you.

^f^Manage alcohol and drug use behavior.

^g^Communicate with your doctor or clinic.

^h^Remember to take your medication.

^i^Engage in social networking with other people with similar health conditions as you.

**Table 3:** Multivariate logistic regression (N=708, 58 missing).

Demographic variable		Odds ratio (95% CI)	*P* value
Age		0.959 (0.936-0.982)	<.001
Hispanic		2.313 (0.920-5.814)	.07
**Race**				
	White	1	—
	Black/African American	1.011 (0.592-1.727)	.97
	Other race	1.491 (0.458-4.854)	.51
Schooling completed		1.281 (1.027-1.598)	.03
Long-term partnership		0.905 (0.523-1.565)	.72
**Sexual orientation**			
	Heterosexual	1	—
	Gay or lesbian	0.798 (0.460-1.385)	.42
	Other sexual preference	0.640 (0.329-1.245)	.19
**Employment status**				
	Employed	1	—
	Unable to work/disabled	0.296 (0.145-0.606)	<.001
	Unemployed	0.736 (0.319-1.696)	.47

## Discussion

### Principal Results

This study found that a vast majority of respondents were interested in using a free mobile phone app that supports HIV self-management. In addition, respondents expressed a strong preference for app functions that could help identify relevant health services, enhance communication with health care providers, provide tips to improve health based on personalized information, supply reminders to take medication, and enable social networking with individuals with similar health conditions. Respondents who were younger and better educated were more likely to express interest in using a phone app for HIV self-management. Conversely, respondents who were disabled or unable to work were significantly less likely to express interest in using a free mobile phone app for HIV self-management. Finally, although Hispanic participants were more than twice as likely to be interested in using a free mobile phone app for HIV self-management than non-Hispanic participants, the association was not statistically significant.

### Limitations

This study provides important insights into PLWH’s level of interest in using a free mobile phone app to support HIV self-management. However, there are limitations to this study. First, the measures are all self-reported by PLWH. This may increase social desirability bias, which occurs when respondents simply provide answers that will be viewed favorably by others. However, this effect was minimized through the use of anonymous surveys. Second, other resources for HIV self-management were not examined in this study. Thus, it is unclear whether respondents who lacked interest in using a free mobile phone app used other resources to self-manage their disease and whether these resources affect their level of interest. However, because a vast majority (approximately 86%) of respondents expressed interest in using a free mobile phone app to support HIV self-management, it is unlikely that this potential limitation had a strong effect on the study.

### Comparison With Prior Work

While little previous work exists examining interest in using free mobile phone apps to support HIV self-management among PLWH, several studies have examined common barriers to HIV self-management in this population. PLWH frequently report barriers related to accessing appropriate medical care, navigating complex medication regimens, and discussing their self-management challenges with providers comfortable with an HIV diagnosis [17]. This study found consistently that a majority of participants were interested in a free mobile phone app that could help address these barriers and strongly supported app functions that aimed to enhance communication with their doctor or clinic and help to identify relevant health care services. Furthermore, respondents liked app functions that could provide personalized tips to improve their health and supply reminders to take medication. These functions can help with the significant challenge of managing complex HIV treatment and medication regimens. Finally, other common barriers to HIV self-management are stigma and lack of social support [17,18]. To address these barriers, around two-thirds of respondents in this study expressed interest in phone app functions that enable social networking with individuals with similar health conditions and provide them with social support with less fear of experiencing stigma. Consistent with findings in this study, previous research has found that people who are younger and better educated are more likely to use phone apps to seek health information and self-manage their disease [17,26-28]. However, for the first time, this study documented that respondents who were unable to work or disabled were significantly less likely to be interested in using a free mobile phone app that supports HIV self-management. This may be because people with disabilities are less likely to own a smartphone and are more likely to report negative experiences with mHealth apps, including feeling overwhelmed by information or unable to find what they need compared to those who are not disabled [29]. It is possible that respondents who were disabled or unable to work were less likely to be interested in using a free mobile app that supports HIV self-management because of previous negative experiences with similar technologies. This highlights the importance of considering issues with accessibility of mobile phone apps for a wide range of people. Indeed, prior research indicates that when mHealth apps are accessible, people with disabilities are avid consumers of health-related technology [29]. Thus, more research is needed to identify the unique needs, barriers, and facilitators of PLWH who are unable to work or disabled to facilitate adoption of technology that can help them effectively self-manage their disease.

### Conclusions

This study revealed that PLWH have a high level of interest in using a free mobile phone app to self-manage their disease. Our findings can be used to inform the development of a mobile phone app that improves PLWH’s ability to self-manage HIV as well as access health care services, communicate with health care providers, and network with individuals with similar conditions. The unique needs of PLWH who are disabled or unable to work should be considered in the adoption and development of technology-based self-management tools and interventions.

## Abbreviations

ART: antiretroviral therapy
HIV: human immunodeficiency virus
NIH: National Institutes of Health
OR: odds ratio
PLWH: people living with human immunodeficiency virus
